# Increasing arterial blood pressure with norepinephrine does not improve microcirculatory blood flow: a prospective study

**DOI:** 10.1186/cc7922

**Published:** 2009-06-17

**Authors:** Arnaldo Dubin, Mario O Pozo, Christian A Casabella, Fernando Pálizas, Gastón Murias, Miriam C Moseinco, Vanina S Kanoore Edul, Fernando Pálizas, Elisa Estenssoro, Can Ince

**Affiliations:** 1 Servicio de Terapia Intensiva, Sanatorio Otamendi y Miroli, Azcuénaga 870, Buenos Aires C1115AAB, Argentina; 2 Cátedra de Farmacología Aplicada, Facultad de Ciencias Médicas, Universidad Nacional de La Plata, 60 y 120, La Plata 1900, Argentina; 3 Servicio de Terapia Intensiva, Clínica Bazterrica, Juncal 3002, Buenos Aires C1425AYN, Argentina; 4 Servicio de Terapia Intensiva, Hospital San Martín, 1 y 70, La Plata 1900, Argentina; 5 Translational Physiology, Academic Medical Center, University of Amsterdam, Meibergdreef 9, Amsterdam 1105 AZ, The Netherlands

## Abstract

**Introduction:**

Our goal was to assess the effects of titration of a norepinephrine infusion to increasing levels of mean arterial pressure (MAP) on sublingual microcirculation.

**Methods:**

Twenty septic shock patients were prospectively studied in two teaching intensive care units. The patients were mechanically ventilated and required norepinephrine to maintain a mean arterial pressure (MAP) of 65 mmHg. We measured systemic hemodynamics, oxygen transport and consumption (DO_2 _and VO_2_), lactate, albumin-corrected anion gap, and gastric intramucosal-arterial PCO_2 _difference (ΔPCO_2_). Sublingual microcirculation was evaluated by sidestream darkfield (SDF) imaging. After basal measurements at a MAP of 65 mmHg, norepinephrine was titrated to reach a MAP of 75 mmHg, and then to 85 mmHg. Data were analyzed using repeated measurements ANOVA and Dunnett test. Linear trends between the different variables and increasing levels of MAP were calculated.

**Results:**

Increasing doses of norepinephrine reached the target values of MAP. The cardiac index, pulmonary pressures, systemic vascular resistance, and left and right ventricular stroke work indexes increased as norepinephrine infusion was augmented. Heart rate, DO_2 _and VO_2_, lactate, albumin-corrected anion gap, and ΔPCO_2 _remained unchanged. There were no changes in sublingual capillary microvascular flow index (2.1 ± 0.7, 2.2 ± 0.7, 2.0 ± 0.8) and the percent of perfused capillaries (72 ± 26, 71 ± 27, 67 ± 32%) for MAP values of 65, 75, and 85 mmHg, respectively. There was, however, a trend to decreased capillary perfused density (18 ± 10,17 ± 10,14 ± 2 vessels/mm^2^, respectively, ANOVA *P *= 0.09, linear trend *P *= 0.045). In addition, the changes of perfused capillary density at increasing MAP were inversely correlated with the basal perfused capillary density (R^2 ^= 0.95, *P *< 0.0001).

**Conclusions:**

Patients with septic shock showed severe sublingual microcirculatory alterations that failed to improve with the increases in MAP with norepinephrine. Nevertheless, there was a considerable interindividual variation. Our results suggest that the increase in MAP above 65 mmHg is not an adequate approach to improve microcirculatory perfusion and might be harmful in some patients.

## Introduction

Septic shock is characterized by severe vasodilation and hypotension refractory to aggressive fluid resuscitation [1]. Despite the normalization of cardiac output, evidence of tissue hypoperfusion is frequently present. Accordingly, organ dysfunctions usually develop despite normal or increased oxygen transport (DO_2_). Microcirculatory alterations could be an underlying explanation for these findings [2]. Experimental models of resuscitated septic shock show that microvascular perfusion is altered despite the normalization of systemic and regional hemodynamics [3]. In addition, septic patients systematically exhibit severe disorders in sublingual microcirculation that are strongly associated with organ failures and outcome [4,5]. The ability to improve sublingual microcirculation has also been related to survival [5]. Moreover, sublingual perfusion might be enhanced by different therapeutic strategies that include the use of vasoactive drugs [6,7]. In this way, improving microcirculation might be an important goal in the resuscitation of patients with septic shock.

An approach to improve microcirculation is to increase the perfusion pressure. When the mean arterial pressure (MAP) decreases below an autoregulatory threshold of about 60 to 65 mmHg, organ perfusion becomes pressure dependent [8]. Nevertheless, intravascular thrombosis and vasoconstrictor mediators, along with regional deficiencies in nitric oxide production, could alter vascular reactivity and shift the autoregulatory threshold to higher values [9,10]. Consequently, the increase in MAP could improve tissue perfusion. Clinical studies, however, have shown that the elevation in MAP beyond 65 mmHg fails to increase systemic oxygen metabolism, skin microcirculatory blood flow, urine output, splanchnic perfusion, or renal function [11,12]. The experimental evidence regarding this issue, however, is controversial [13-17].

Our goal was to assess the effects of titration of a norepinephrine infusion to increasing levels of MAP on sublingual microcirculation. We hypothesized that the increase in MAP from 65 to 75 mmHg, and then to 85 mmHg does not improve sublingual microcirculatory blood flow. At the time of submission of this manuscript, a very similar study was published [18], which reported that escalating doses of norepinephrine in septic patients increased DO_2 _and tissue oxygenation, and were not associated with significant changes in preexisting sublingual microvascular alterations. The results of our study confirm these previous findings, but suggest that the individual responses are related to the basal microcirculatory condition.

## Materials and methods

The protocol was approved by the Institutional Review Boards of Sanatorio Otamendi and Clínica Bazterrica. Informed consent was obtained from the next of kin of all patients admitted to the study.

### Setting

This study was conducted in two teaching intensive care units.

### Patients

The study population included 20 septic shock patients [19] requiring norepinephrine despite adequate fluid resuscitation to maintain a MAP of 65 mmHg or higher (Table 1). They were mechanically ventilated in controlled mode and received infusions of midazolam and fentanyl. All patients had a systemic arterial catheter and a pulmonary artery catheter inserted. A tonometric nasogastric tube was placed into the stomach (TRIP NGS Catheter, Tonometrics, Worcester, MA, USA), after which radiographic confirmation of catheter position was obtained. All patients received intravenous ranitidine. The clinical characteristics of the patients are presented in Table 1.

**Table 1:** Clinical and epidemiological characteristics of the patients

**Age, years**	72 ± 12
**Gender, % male**	55
**SOFA score**	9.8 ± 2.8
**APACHE II score**	24.4 ± 5.4
**APACHE II predicted risk mortality**	50.8 ± 17.5
**Actual mortality, %**	50
**Source of sepsis, n (%)**	
**Pneumonia**	9 (45)
**Intra-abdominal infection**	3 (15)
**Primary bacteremia**	3 (15)
**Endovascular infection**	2 (10)
**Cellulitis**	2 (10)
**Urinary tract infection**	1 (5)
**Fluid balance on the previous day, ml**	4592 ± 3156
**Fluid administration on the previous day, ml**	6183 ± 2601

APACHE = Acute Physiology and Chronic Health Evaluation; SOFA = Sepsis-related Organ Failure Assessment.

### Measurements and derived calculations

Serial measurements of heart rate, MAP, mean arterial pulmonary pressure, pulmonary artery occlusion pressure, and central venous pressure were performed. Transducers were referenced to the midaxillary line and all pressures were taken at end-expiration. Cardiac output was measured by thermodilution using three injections of saline solution (10 cc) at room temperature.

Arterial, mixed venous, and central venous blood samples were analyzed for gases, hemoglobin, and oxygen saturation (AVL OMNI 9, Roche Diagnostics, Graz, Austria). Sodium (Na), potassium (K) and chloride (Cl) ions (selective electrode ion, AEROSET, Abbott Laboratories, Abbott Park, IL, USA), albumin (Bromcresol-sulfonphthaleinyl), and lactate (selective electrode ion, AVL OMNI 9) were measured in arterial blood samples. The albumin-corrected anion gap was calculated [20] as: 



Derived hemodynamic and DO_2 _variables were calculated according to standard formulae.

Intramucosal partial pressure of carbon dioxide (PCO_2_) was measured with a tonometer using an automated air tonometry system (Tonocap; Datex Ohmeda, Helsinki, Finland). Its value was used to calculate the intramucosal-arterial PCO_2 _difference (ΔPCO_2_).

### Microvideoscopic measurements and analysis

The microcirculatory network was evaluated in the sublingual mucosa using a sidestream dark field (SDF) imaging device (Microscan^®^, MicroVision Medical, Amsterdam, Netherlands) [21].

Different cautions and steps were followed to obtain images of adequate quality and to ensure good reproducibility. Video acquisition and image analyses were performed by well-trained researchers (AD, MOP and VSKE). After gentle removal of saliva by isotonic-saline-drenched gauze, steady images of at least 20 seconds were obtained while avoiding pressure artifacts using a portable computer and an analog/digital video converter (ADVC110, Canopus Co, San Jose, CA, USA). Video clips were stored as AVI files to allow computerized frame-by-frame image analysis. SDF images were acquired from at least five different sites. Adequate focus and contrast adjustment were verified, and images of poor quality were discarded. The entire sequence was used to characterize the semi-quantitative characteristics of microvascular blood flow, particularly the presence of stopped or intermittent flow.

Video clips were analyzed blindly and randomly using different approaches. First, we used a previously validated semi-quantitative score [22]. It distinguishes between no flow (0), intermittent flow (1), sluggish flow (2), and continuous flow (3) [22]. A value was assigned to each individual vessel. The overall score, called the microvascular flow index (MFI), is the average of the individual values. For each patient, the values from five to eight videos were averaged. In addition, vascular density was quantified as the number of vessels per mm^2^. To determine heterogeneity of perfusion in each territory, the flow heterogeneity index was calculated as the highest MFI minus the lowest MFI divided by the mean MFI [23]. These quantifications of flow were made per group of vessel diameter: small (capillaries), 10 to 20 μm; medium, 21 to 50 μm; and large, 51 to 100 μm. Finally, the percentage of perfused vessels and the total and capillary perfused vascular densities were calculated [4,24]. The percentage of perfused vessels was calculated as the number of vessels with flow 2 and 3 divided by the total number of vessels multiplied by 100.

### Study protocol

After fluid resuscitation failed to improve MAP, a norepinephrine infusion was adjusted to reach a MAP of 65 mmHg in all patients. After a period of at least two hours in which the requirement of norepinephrine to maintain a MAP of 65 mmHg remained unchanged, the measurements were performed. Norepinephrine was then titrated to reach a MAP of 75 mmHg. After 30 minutes at this MAP, new measurements were taken. Finally, norepinephrine infusion was increased to achieve a MAP of 85 mmHg and, after 30 minutes at this MAP, a final set of measurements were taken.

No additional sedation, antipyretics or vasoactive drugs were administered during the study period. The infusions of midazolam and fentanyl were kept constant at rates of 0.99 ± 0.22 mg/kg/hour and 0.82 ± 0.20 μg/kg/hour, respectively.

### Analysis of data

After showing a normal distribution, data were analyzed using repeated measurements analysis of variance (ANOVA) and Dunnett test. Linear trends between the different variables and increasing levels of MAP were calculated [25]. A *P *< 0.05 was considered significant. Data are showing as mean ± standard deviation.

## Results

### Effects on hemodynamic and oxygen transport variables

Increasing doses of norepinephrine induced the target values of MAP. Cardiac index and pulmonary pressures increased as norepinephrine infusion was augmented. Heart rate, DO_2 _and oxygen consumption remained unchanged (Table 2).

**Table 2:** Changes in hemodynamic, oxygen transport, and tonometric variables as mean arterial pressure was increased from 65 mmHg to 85 mmHg with norepinephrine

	**Mean arterial blood pressure**			**ANOVA**	**Linear trend**
			
	**65 mmHg**	**75 mmHg**	**85 mmHg**	***P***	***P***
**Norepinephrine doses (μg/kg/min)**	0.48 ± 0.43	0.65 ± 0.68*	0.74 ± 0.67*	< 0.0001	< 0.0001
**Heart rate (beats/min)**	94 ± 21	92 ± 18	93 ± 18	0.59	0.43
**Mean arterial blood pressure (mmHg)**	65 ± 2	76 ± 2*	85 ± 2*	< 0.0001	< 0.0001
**Mean pulmonary artery pressure (mmHg)**	28 ± 7	30 ± 7*	30 ± 7*	< 0.0001	< 0.0001
**Pulmonary artery occlusion pressure (mmHg)**	14 ± 4	15 ± 4	16 ± 4	0.06	0.02
**Central venous pressure (mmHg)**	11 ± 4	12 ± 4	12 ± 4	0.18	0.47
**Cardiac index (l/min/m^2^)**	2.98 ± 0.99	3.11 ± 1.07	3.23 ± 1.02*	0.0002	< 0.0001
**Oxygen transport (ml/min/m^2^)**	366 ± 137	379 ± 145	383 ± 166	0.53	0.28
**Oxygen consumption (ml/min/m^2^)**	100 ± 33	91 ± 31	90 ± 40	0.61	0.63
**Intramucosal-arterial pCO_2 _(mmHg)**	15 ± 15	16 ± 18*	16 ± 18	0.03	0.06

* *P *< 0.05 vs. basal (Dunnett post hoc test after repeated measures ANOVA).

ANOVA = analysis of variance; pCO_2 _= partial pressure of carbon dioxide.

### Effects on lactate and acid-base parameters

Arterial lactate levels were stable. Venous oxygen saturations and pressures increased while other acid-base variables were unmodified (Table 3).

**Table 3:** Changes in arterial lactate, hemoglobin, blood gases and oxygen saturations as mean arterial pressure was increased from 65 mmHg to 85 mmHg with norepinephrine

	**Mean arterial blood pressure**			**ANOVA**	**Linear trend**
			
	**65 mmHg**	**75 mmHg**	**85 mmHg**	***P***	***P***
**Arterial lactate (mmol/L)**	2.6 ± 2.8	2.4 ± 2.7	2.5 ± 2.7	0.27	0.32
**Hemoglobin (g%)**	9.6 ± 2.3	9.6 ± 2.4	9.6 ± 2.3	0.76	0.79
**Arterial pH**	7.26 ± 0.11	7.26 ± 0.11	7.26 ± 0.11	0.44	0.29
**Arterial PCO_2 _(mmHg)**	39 ± 10	39 ± 10	40 ± 11	0.73	0.57
**Arterial PO_2 _(mmHg)**	112 ± 48	113 ± 45	108 ± 34	0.39	0.25
**Arterial HCO_3_^- ^(mmol/l)**	18 ± 5	18 ± 5	18 ± 5	0.50	0.43
**Arterial oxygen saturation**	0.96 ± 0.02	0.96 ± 0.02	0.96 ± 0.03	0.67	0.44
**Mixed venous pH**	7.23 ± 0.11	7.24 ± 0.10	7.24 ± 0.10	0.78	0.49
**Mixed venous PCO_2 _(mmHg)**	45 ± 11	45 ± 11	45 ± 11	0.90	0.69
**Mixed venous PO_2 _(mmHg)**	42 ± 7	43 ± 7	44 ± 8*	0.04	0.02
**Mixed venous HCO_3_^- ^(mmol/l)**	19 ± 5	19 ± 5	19 ± 5	0.18	0.08
**Mixed venous oxygen saturation**	0.70 ± 0.08	0.72 ± 0.08*	0.73 ± 0.07*	0.01	0.005
**Central venous oxygen saturation**	0.74 ± 0.08	0.76 ± 0.08*	0.77 ± 0.08*	0.01	0.004
**Arterial anion gap (mmol/L)**	18 ± 6	19 ± 6	20 ± 7	0.16	0.06

* *P *< 0.05 vs. basal (Dunnett *post hoc *test after repeated measures ANOVA).

ANOVA = analysis of variance; HCO_3 _= bicarbonate; pCO_2 _= partial pressure of carbon dioxide; pO_2 _= partial pressure of oxygen.

### Effects on gastric tonometry

ΔPCO_2 _did not change throughout the study (Table 2).

### Effects on sublingual microcirculation

**Figure 1:**
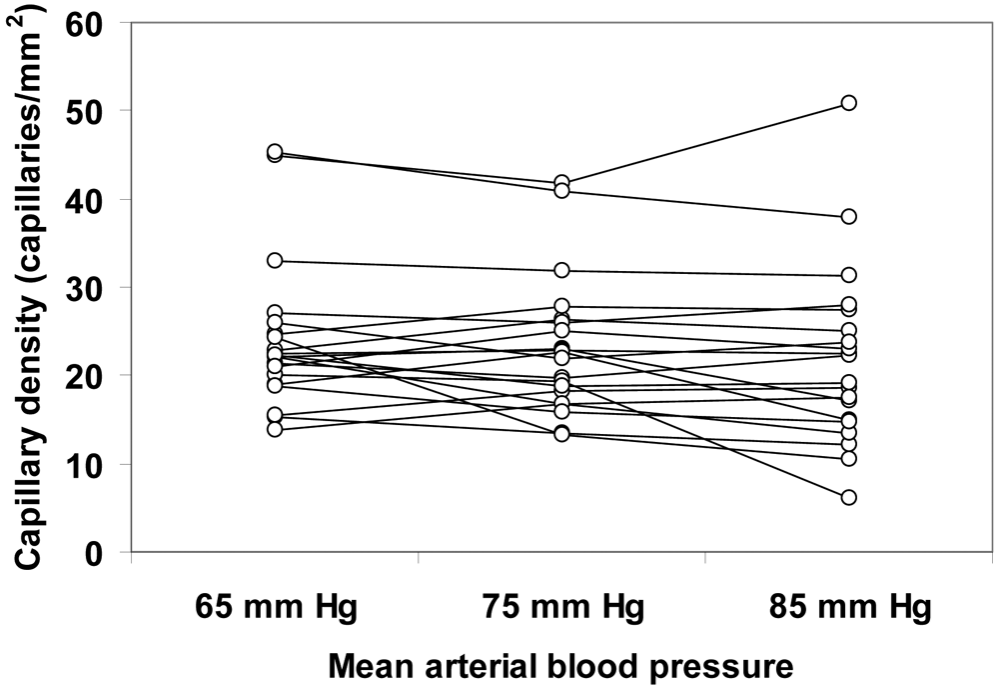
Individual behavior of the sublingual capillary density. Results are shown as the mean arterial pressure was increased from 65 mmHg to 85 mmHg with norepinephrine.

**Figure 2:**
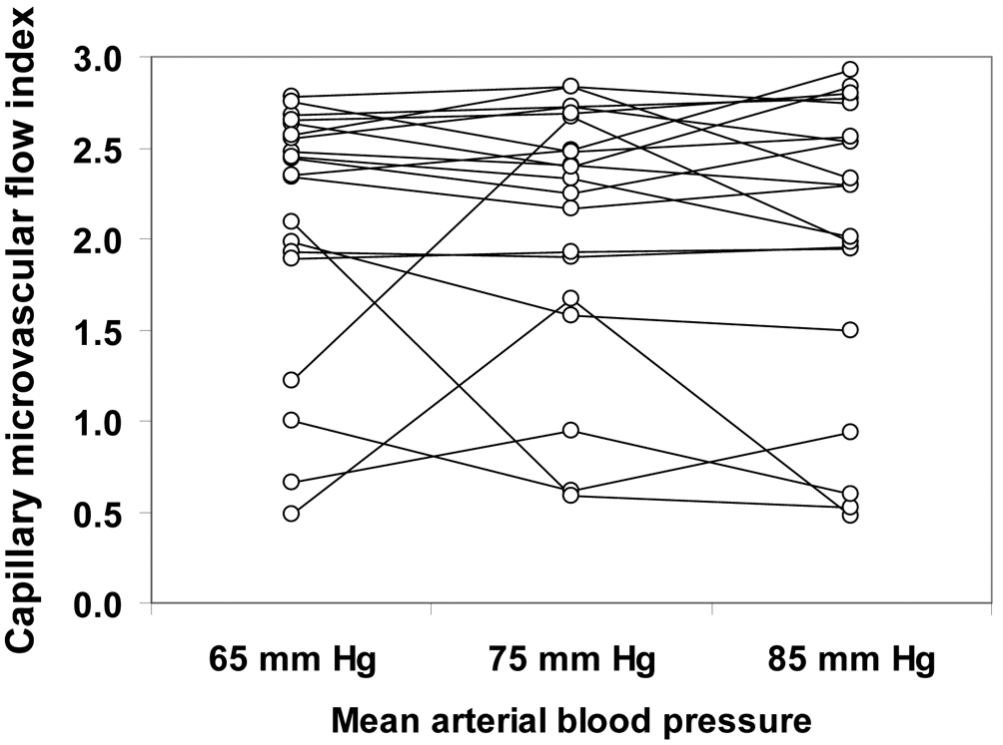
Individual behavior of sublingual capillary microvascular flow index. Results are shown as the mean arterial pressure was increased from 65 mmHg to 85 mmHg with norepinephrine.

**Figure 3:**
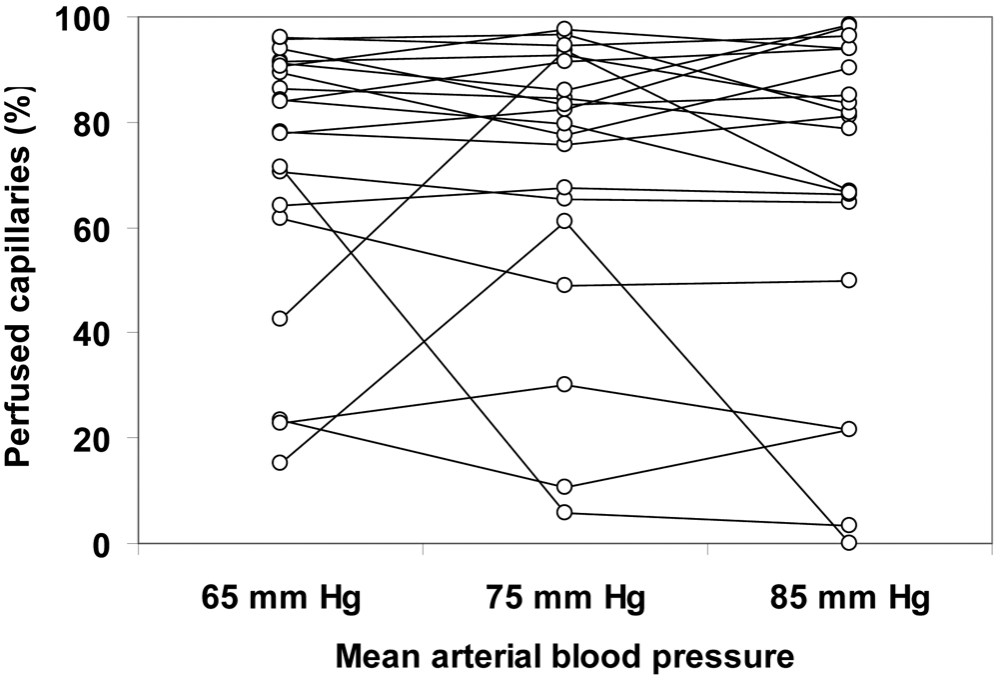
Individual behavior of sublingual percentage of perfused capillaries. Results are shown as the mean arterial pressure was increased from 65 mmHg to 85 mmHg with norepinephrine.

**Figure 4:**
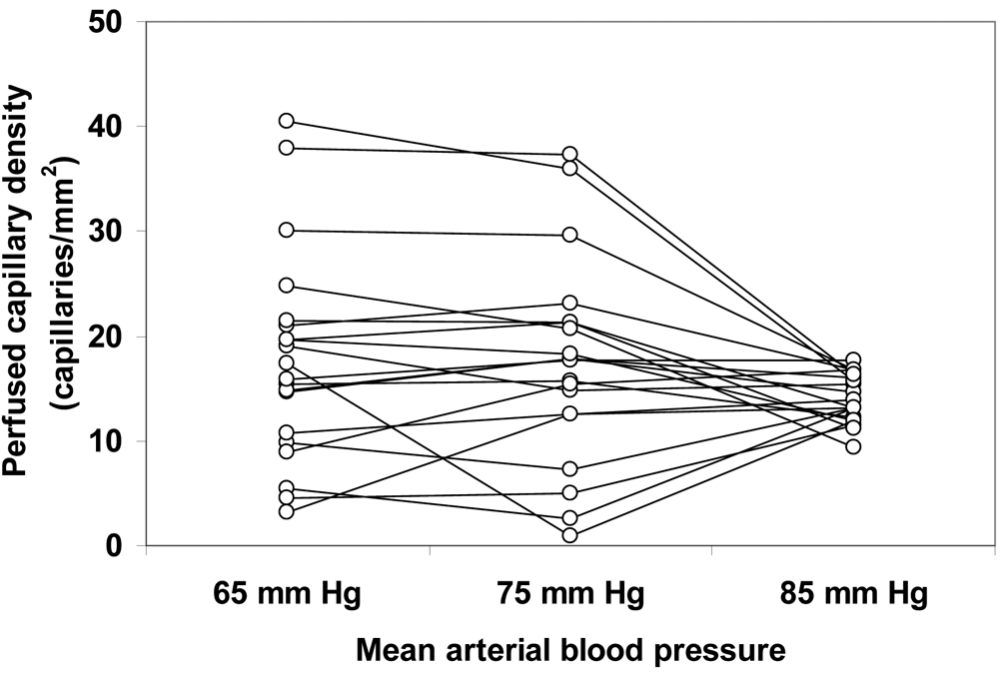
Individual behavior of sublingual perfused capillary density. Results are shown as the mean arterial pressure was increased from 65 mmHg to 85 mmHg with norepinephrine.

**Figure 5:**
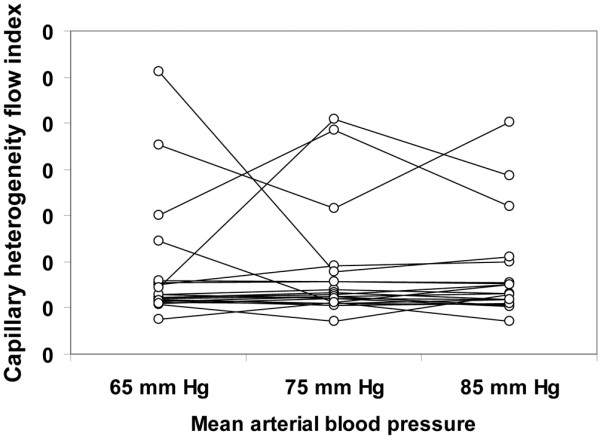
Individual behaviour of sublingual capillary heterogeneity flow index. Results are shown as the mean arterial pressure was increased from 65 mmHg to 85 mmHg with norepinephrine.

**Figure 6:**
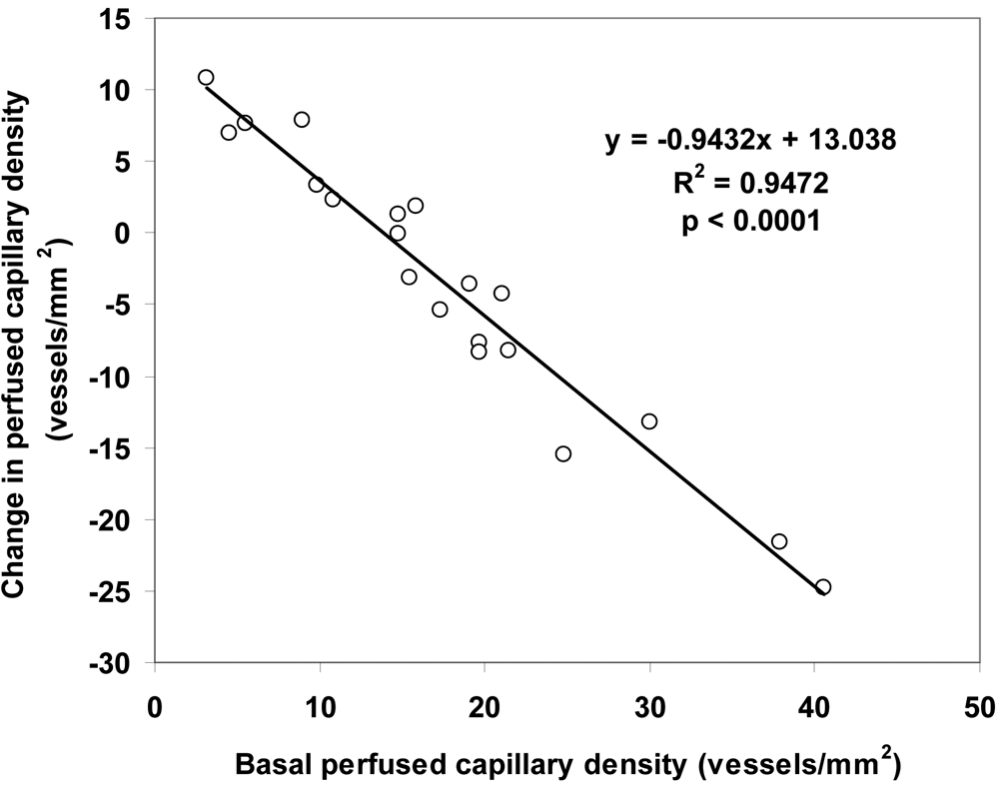
Relationship between the changes of perfused capillary density, when mean arterial pressure (MAP) was increased from the baseline to a MAP of 85 mmHg, with the basal perfused capillary density at a MAP of 65 mmHg.

Although the total vascular density was not significantly altered, there was a trend to a decreased capillary density (ANOVA *P *= 0.09, linear trend *P *= 0.03; Table 4). The MFI and the percentage of perfused vessels were unchanged in the different types of vessels at increasing MAP values. The total perfused vascular density was unmodified for MAP values of 65, 75, and 85 mmHg (38 ± 14, 37 ± 15, 37 ± 4 vessels/mm^2^, respectively, ANOVA *P *= 0.94, linear trend *P *= 0.76); however, there was a trend to a decreased perfused capillary density (18 ± 10, 17 ± 10, 14 ± 2 vessels/mm^2^, respectively, ANOVA *P *= 0.09, linear trend *P *= 0.045). The heterogeneity flow index also remained unchanged (Table 4). The individual behaviour of capillary density, capillary MFI, percentage of perfused capillaries, perfused capillary density and capillary heterogeneity flow index are depicted in Figures 1 to 5. There was, however, considerable interindividual variability. In particular, there was a strong linear relationship between the changes of perfused capillary density, when MAP was increased from baseline to 85 mmHg, with the basal perfused capillary density at a MAP of 65 mmHg (Figure 6).

**Table 4:** Changes in microvascular variables as mean arterial pressure was increased from 65 mmHg to 85 mmHg with norepinephrine

	**Mean arterial blood pressure**			**ANOVA**	**Linear trend**
			
	**65 mmHg**	**75 mmHg**	**85 mmHg**	***P***	**P**
Vascular density (vessels/mm^2^)					
Large diameter vessels	11 ± 1	10 ± 3	10 ± 3	0.81	0.61
Medium diameter vessels	15 ± 3	16 ± 4	16 ± 4	0.82	0.53
Small diameter vessels	24 ± 8	23 ± 8	22 ± 1	0.09	0.03
Microvascular flow index					
Large diameter vessels	2.3 ± 0.6	2.3 ± 0.8	2.2 ± 0.8	0.34	0.16
Medium diameter vessels	2.2 ± 0.7	2.2 ± 0.7	2.1 ± 0.9	0.79	0.52
Small diameter vessels	2.1 ± 0.7	2.2 ± 0.7	2.0 ± 0.8	0.69	0.47
Perfused vessels (%)					
Large diameter vessels	82 ± 21	80 ± 28	87 ± 6	0.46	0.40
Medium diameter vessels	77 ± 27	77 ± 29	77 ± 6	0.98	1.00
Small diameter vessels	72 ± 26	71 ± 27	67 ± 32	0.55	0.38
Total vessels	75 ± 25	75 ± 27	76 ± 4	0.92	0.73
Heterogeneity flow index					
Large diameter vessels	1.0 ± 0.5	1.3 ± 1.2	1.5 ± 1.4	0.07	0.02
Medium diameter vessels	1.6 ± 1.6	1.5 ± 1.4	1.7 ± 1.2	0.86	0.78
Small diameter vessels	1.8 ± 1.3	1.8 ± 1.2	1.7 ± 1.1	0.97	0.80

ANOVA = analysis of variance.

## Discussion

The main finding of this study is that the increase in MAP with norepinephrine failed to improve sublingual microcirculation, or any other variable related to perfusion as arterial lactate, anion gap, ΔPCO_2_, and parameters of oxygen metabolism. Despite a trend to decreased total and perfused capillary density, there were considerable variations in the interindividual responses that seem to depend on the basal condition of the microcirculation.

The goal of vasopressor therapy is to improve tissue perfusion pressure, while avoiding excessive vasoconstriction. Marik and Mohedin showed that an infusion of norepinephrine titrated to increase the MAP to more than 75 mmHg improved intramucosal pH [26]. Martin and colleagues [27] and Desjars and colleagues [28] reported significant increases in urine output and improvements in renal function in septic shock. Nevertheless, in these studies [26-28] the initial MAP was below 60 mmHg, a value that is most likely beyond the lower limit of autoregulation. On the other hand, Deruddre and colleagues showed that increasing MAP from 65 to 75 mmHg with norepinephrine in patients with septic shock increased urinary output and decreased renal vascular resistance [29].

Our study is consistent with the results from LeDoux and colleagues [11] and Bourgoin and colleagues [12]. In these studies, the lack of change in any perfusion variable over a range of 20 mmHg in MAP suggests that the patients were within their autoregulatory range. Jhanji and colleagues have recently demonstrated that increasing doses of norepinephrine resulted in an increase in global DO_2_, and in cutaneous microvascular flow and tissue partial pressure of oxygen (PO_2_) without significant changes in sublingual microcirculation [18]. They also showed, however, that when MAP was augmented from 70 to 90 mmHg, the MFI, proportion of perfused vessels, and perfused vessel density fell by about 10%. The remarkable similarity between the study by Jhanji and colleagues [18] and the current study emphasizes the reproducibility of the techniques and results.

In addition, our results expand previous knowledge by addressing the variation of interindividual responses. In particular, the change in the perfused capillary density was strongly dependent on the basal state of microcirculation. In this way, perfused capillary density improved in patients with an altered sublingual perfusion at baseline, and decreased in patients with preserved basal microvascular perfusion. Sakr and colleagues described a similar microvascular response to red blood cell transfusion [30]. Other studies have also shown that vasopressors could decrease sublingual microcirculation [31,32], suggesting that excessive vasoconstriction might be deleterious to microcirculation.

Our study has several limitations. First, this observational study lacks a control group. Each patient, therefore, served as his/her own control. Second, the number of patients included in this study was small. Despite the sample size, significant changes in hemodynamic variables developed. Conversely, most parameters related to tissue perfusion and oxygenation remained unchanged or had a trend to worsen. Third, the infusion period was short. A longer period might have allowed the appearance of changes not observed in the present study. Nevertheless, most norepinephrine effects on hemodynamics and on tissue perfusion and oxygenation are expected to be evident within a few minutes. In addition, the short half-life of norepinephrine allows a new steady state to be reached in its plasmatic levels a few minutes after a change in the infusion rate [33]. In fact, several hemodynamic changes appeared shortly after each dose modification. Moreover, the infusion period was deliberately kept short, to avoid the background effects of changes in the underlying conditions of the patients. With longer periods of evaluation, a time effect with spontaneous changes of the studied variables related to the natural history of the disease might not be ruled out. Finally, as a different behaviour of microcirculatory beds is a characteristic of sepsis [3,34], this study does not address the response of other territories to increasing MAP.

## Conclusions

In this observational study, patients with septic shock showed severe microcirculatory alterations that failed to improve with the increases in MAP with norepinephrine. Furthermore, linear trend analysis showed reductions in the capillary and in the perfused capillary densities. Nevertheless, interindividual responses could be quite variable and dependent on the basal state of the microcirculation. Our results suggest that the increase in MAP above 65 mmHg is not a straightforward treatment to improve microvascular perfusion. It might be harmful for some patients, while benefiting others. Studies including greater numbers of patients are needed to determine the usefulness of individual titration of vasopressor therapy on sublingual microcirculation.

## Key messages

• Patients with septic shock showed severe microcirculatory abnormalities that an increase in MAP with norepinephrine globally failed to improve.

• The change in the perfused capillary density was strongly dependent on the basal state of the microcirculation. Thus, perfused capillary density improved in patients with an altered sublingual perfusion at baseline, and decreased in patients with preserved basal microvascular perfusion.

## Abbreviations

ANOVA: analysis of variance; DO_2_: oxygen transport; MAP: mean arterial pressure; MFI: microvascular flow index; PCO_2_: partial pressure of carbon dioxide; ΔPCO_2_: gastric intramucosal-arterial PCO_2 _difference; PO_2_: partial pressure of oxygen; SDF: sidestream darkfield.

## Competing interests

CI is Chief Scientific Officer of MicroVision Medical (a university-based company manufacturing sidestream dark field devices) and holds patents and stock related to SDF imaging. The remaining authors have not disclosed any potential conflicts of interest.

## Authors' contributions

AD designed the study, performed the statistical analysis, and drafted the manuscript. MOP and VSKE were involved in the analysis of the videos. AD, MOP, CAC, FPJr, GM, MCM, and FP made substantial contributions to acquisition of data. EE and CI made substantial contributions to analysis and interpretation of data, and were involved in drafting the manuscript and revising it critically for important intellectual content. All authors read and approved the final manuscript.
